# Glycerophosphoinositol Promotes Apoptosis of Chronic Lymphocytic Leukemia Cells by Enhancing Bax Expression and Activation

**DOI:** 10.3389/fonc.2022.835290

**Published:** 2022-03-22

**Authors:** Gioia Boncompagni, Alessia Varone, Vanessa Tatangelo, Nagaja Capitani, Federica Frezzato, Andrea Visentin, Livio Trentin, Daniela Corda, Cosima T. Baldari, Laura Patrussi

**Affiliations:** ^1^ Department of Life Sciences, University of Siena, Siena, Italy; ^2^ Institute of Endocrinology and Experimental Oncology “G. Salvatore”, National Research Council, Naples, Italy; ^3^ Hematology and Clinical Immunology Unit, Department of Medicine, University of Padua, Padua, Italy; ^4^ Department of Biomedical Sciences, National Research Council, Rome, Italy

**Keywords:** CLL, apoptosis, Bax, glycerophosphoinositol, SHP-1

## Abstract

An imbalance in the expression of pro- and anti-apoptotic members of the Bcl-2 family of apoptosis-regulating proteins is one of the main biological features of CLL, highlighting these proteins as therapeutic targets for treatment of this malignancy. Indeed, the Bcl-2 inhibitor Venetoclax is currently used for both first-line treatment and treatment of relapsed or refractory CLL. An alternative avenue is the transcriptional modulation of Bcl-2 family members to tilt their balance towards apoptosis. Glycerophosphoinositol (GroPIns) is a biomolecule generated from membrane phosphoinositides by the enzymes phospholipase A_2_ and lysolipase that pleiotropically affects key cellular functions. Mass-spectrometry analysis of GroPIns interactors recently highlighted the ability of GroPIns to bind to the non-receptor tyrosine phosphatase SHP-1, a known promoter of Bax expression, suggesting that GroPIns might correct the Bax expression defect in CLL cells, thereby promoting their apoptotic demise. To test this hypothesis, we cultured CLL cells in the presence of GroPIns, alone or in combination with drugs commonly used for treatment of CLL. We found that GroPIns alone increases Bax expression and apoptosis in CLL cells and enhances the pro-apoptotic activity of drugs used for CLL treatment in a SHP-1 dependent manner. Interestingly, among GroPIns interactors we found Bax itself. Short-term treatments of CLL cells with GroPIns induce Bax activation and translocation to the mitochondria. Moreover, GroPIns enhances the pro-apoptotic activity of Venetoclax and Fludarabine in CLL cells. These data provide evidence that GroPIns exploits two different pathways converging on Bax to promote apoptosis of leukemic cells and pave the way to new studies aimed at testing GroPIns in combination therapies for the treatment of CLL.

## Introduction

Chronic lymphocytic leukemia (CLL), the most common lymphoid malignancy in Western countries, is characterized by the accumulation of monoclonal CD5^+^ B cells in peripheral blood, bone marrow and secondary lymphoid organs (1). Although the clinical course is highly variable, the most conserved feature of CLL is the extended survival of malignant B cells, which has been associated to defects in the apoptotic machinery (1, 2).

Alterations in the expression of pro-survival and pro-apoptotic members of the B-cell leukemia/lymphoma-2 (Bcl-2) family of apoptosis-regulating proteins is a hallmark of CLL and a key intrinsic factor underlying the longevity of CLL cells (1, 2). Increased expression of pro-survival members such as Bcl-2 and Mcl-1 (3, 4), concomitant with impaired expression of pro-apoptotic members such as Bax and Bak (5), tilts the finely regulated balance towards survival, leading to the accumulation of long-lived neoplastic cells that further acquire stroma-derived survival signals during their transit through secondary lymphoid organs (2, 6). It is therefore not surprising that restoring the Bcl-2 family balance has been pinpointed as strategy for overcoming the apoptosis defects of CLL cells, as witnessed by the recent approval of the Bcl-2 selective inhibitor Venetoclax for CLL treatment (7, 8). This effect is also elicited by chemotherapeutic drugs such as the fluorinated nucleotide analog Fludarabine, which affects the Bcl-2 family balance by indirectly promoting both expression and activation of Bax (9, 10). As opposed to Bcl-2, no drugs that specifically target Bax to enhance its expression or activation have been as yet developed (11).

Glycerophosphoinositols (GPIs) are water-soluble bioactive phospholipid derivatives of increasing interest as intracellular and paracrine mediators of eukaryotic cell functions. Generated from membrane phosphoinositides by the phospholipase cPLA_2_α, GPIs have diverse effects in a variety of cell types (12, 13). The most representative compound of the family is glycerophosphoinositol (GroPIns), a ubiquitous component of mammalian cells that participates in cell proliferation and survival in response to extracellular stimuli (14). When added exogenously, GroPIns elicits pharmacological effects relevant to both inflammatory responses and tumor spreading. In human blood monocytes GroPIns counteracts the LPS-induced proinflammatory and prothrombotic responses, inhibiting TLR4 signaling and leading to a decrease in the NF-κB-dependent transcription of inflammatory genes (15). GroPIns has also been recently found to reduce the invasive potential of melanoma cells through its ability to interact with and regulate the non-receptor tyrosine phosphatase Src homology region 2 domain-containing phosphatase-1 (SHP-1) (16, 17). GroPIns interaction with SHP-1 facilitates SHP-1 localization to invadopodia where it dephosphorylates cortactin, with subsequent impaired invadopodia function and hampered metastasis of melanoma cells both *in vitro* and *in vivo* (17).

Mainly expressed in hematopoietic and epithelial cells, the tyrosine phosphatase SHP-1 is a negative regulator of signaling pathways leading to cell proliferation, differentiation, survival and adhesion (18). Its dephosphorylating activity makes it a key regulator of cancer progression. Both expression and activity of SHP-1 are impaired in a number of cancer cell lines and tissues (19–21). Several pharmacological drugs used for cancer treatment enhance SHP-1 expression, which in turn downregulates aberrantly activated tyrosine kinase-dependent signaling pathways (22). The involvement of SHP-1 in cancer progression is also supported by evidence that SHP-1 promotes cancer cell apoptosis (23, 24) by enhancing the expression of Bax (23, 25). Although its expression levels are unaffected in CLL cells, SHP-1 activity is inhibited as a result of phosphorylation of the inhibitory residue Ser591 (26), making it an interesting molecular target for the treatment of this disease.

Here we asked whether GroPIns affects CLL cell apoptosis. We show that GroPIns exploits its SHP-1 modulating activity to promote CLL cell apoptosis by enhancing Bax expression. Moreover, we show that GroPIns directly interacts with Bax, rapidly promoting its activation and recruitment to the mitochondria. Hence GroPIns promotes CLL cell apoptosis by regulating the expression and activation of Bax through different pathways, highlighting the potential exploitability of this glycerophospholipid to overcome the apoptosis defects of CLL cells.

## Materials and Methods

### Cells, Antibodies and Reagents

Peripheral blood samples were collected from 40 treatment-naive CLL patients. Diagnosis of CLL was made according to international workshop on CLL (iwCLL) 2008 criteria (27). The immunophenotypic analysis of lymphocytes obtained from peripheral blood of CLL patients was performed by flow cytometry. All patients expressed the typical phenotypic profile according to standard criteria for CLL diagnosis and were positive for CD19, CD5, CD23 and CD200. Flow cytometric plots of a representative CLL patient are shown in **Supplementary Figure 1**. Mutational *IGHV* status was assessed as reported (28). The main clinical features of CLL patients used in this study are listed in **Supplementary Table 1**. B cells from 24 buffy coats were used as healthy population controls. B cells were purified by negative selection using RosetteSep B-cell enrichment Cocktail (StemCell Technologies, Vancouver, Canada) followed by density gradient centrifugation on Lympholite (Cedarlane Laboratories, The Netherlands), as reported (29). Human HS-5 (30) stromal cells were used for co-culture experiments, as reported (31). Cells were maintained in RPMI (Roswell Park Memorial Institute)-1640 (Merck, #R8758) containing 7.5% Bovine Calf Serum (BCS) (HyClone, #SH30072.03). GroPIns was kindly provided by Euticals S.p.a (Lodi, Italy). GroPIns-Bio was obtained from Echelon Biosciences (Salt Lake City, UT, USA). NSC-87887 (Merck, #565851and Fludarabine (Merck, #F9813) were from Merck. Venetoclax was from Selleck Chemicals (#S8048). His-tagged Bax-α lacking 21 amino acids at the C-terminus (His-BaxΔTM) cloned in the pTrcHis vector (Invitrogen Srl) was a kind gift of Ingram Iaccarino. This construct was expressed in *E. coli* BL21(DE3)/pLysS cells and purified as described (32).

### Cell Treatments, Antibodies and Immunoblots

Treatments with 100 μM GroPIns, 35 μM Fludarabine, 3.5 nM Venetoclax or combination treatments were carried out at 37°C in RPMI 7.5% BCS for the indicated times. Control samples were treated with DMSO (Merck Millipore, #102952). Dose-response and time course experiments of CLL B cells treated with GroPIns are shown in **Supplementary Figure 2**. When required, cells were pretreated at 37°C for 20 min with 50 μM NSC-87887. Cells (5×10^6^ cells/sample) were lysed in 1% (v/v) Triton X-100 in 20 mM Tris-HCl pH 8, 150 mM NaCl, in the presence of a cocktail of protease inhibitors (Calbiochem, #539134) and 0.2 mg/ml Na orthovanadate (Merck, #S6508), resolved by SDS-PAGE and transferred to nitrocellulose (GE Healthcare, #9004-70-0). Immunoblots were carried out using mouse anti-Bax (BD Biosciences, #610982), anti-penta-His (Life Technologies, #P21315) and anti-actin (Millipore, #MAB1501) primary antibodies. Secondary peroxidase-labeled anti-mouse antibodies were from Jackson Immuno-Research (#115-035-146). Labeled antibodies were detected using ECL kit (SuperSignal^®^ West Pico Chemiluminescent Substrate, Thermo Scientific) and scanned immunoblots were quantified using the ImageJ software.

### Intracellular Staining, Apoptosis, TMRM Assays and Flow Cytometry

Cells (2×10^5^ cells/sample) were treated for 20 min in complete medium at 37°C as above, washed with PBS and fixed in 100 μl of fixation buffer (eBiosciences, #420801) for 15 minutes at RT. Cells were then washed with PBS added with 1% BSA (AppliChem PanReac, #A6588) and incubated with 10 μl permeabilization buffer (eBiosciences, #421008) containing either mouse anti-Bax (B-9) (Santa Cruz Biotechnology Inc., #sc-7480) or rabbit anti-phospho-SHP-1 Tyr564 (Cell Signaling, #D11G5) antibodies at RT for 1 h, washed twice in PBS 1% BSA and then incubated with 10 μl permeabilization buffer containing Alexa Fluor anti-mouse-488 (Thermo Fisher Scientific, #A11001) or anti-rabbit-488 (Thermo Fisher Scientific, #A11008) secondary antibodies for 45 min. After washing with PBS 1% BSA, cell pellets were resuspended in 200 μl PBS 1% BSA and subjected to flow cytometric analysis. Early apoptotic cells were quantified by flow cytometric analysis of 1×10^6^ cells stained with FITC-labeled Annexin V (e-Bioscience, #88-8005-74) and Propidium iodide (PI, 20 µg/mL, Biotium, #40017). Mitochondrial membrane potential was measured using the fluorescent probe tetramethylrhodamine methyl ester (TMRM, Molecular Probes Europe BV). Cells (10^6^ cells/sample) were suspended in 200 μl RPMI-1640 w/o phenol Red (Invitrogen srl) added with 25 mM Hepes pH 7.4 and 200 nM TMRM and incubated for 20 min at 37°C. Cells were then added with 500 ng/ml of the calcium ionophore A23187 (Sigma-Aldrich #C7522), incubated for 10 min at 37°C and subjected to flow cytometric analysis. Flow cytometry was carried out using a Guava Millipore cytometer as described (29). Data were analyzed using Flowjo (Tree Star, Inc.).

### Co-Culture Experiments

Stromal cells were seeded on 96-well plates (1.5 × 10^5^ cells/well) in complete culture medium and cultured to confluence. 2 × 10^5^ cells/well CLL cells were added. Cells were co-cultured for 24 h at 37°C in the presence of either Venetoclax or DMSO. Wells were gently washed with RPMI to recover CLL cells, avoiding HS-5 cell detachment from the wells. Samples were stained with either CD19-FITC antibody (Biolegend, #392503) to identify the CLL cell population or with FITC-labeled Annexin V/Propidium iodide to evaluate early apoptotic cells, and analyzed by flow cytometry.

### GroPIns-Bio Pull-Down Assay

GroPIns-Bio pull-down assays were previously described (16). Briefly, Raw 264.7 cells were centrifuged, washed with PBS and re-suspended in lysis buffer supplemented with a protease inhibitor cocktail (Complete Mini EDTA-free, Roche). The cell lysate was kept on a rotating wheel for 30 min at 4°C, centrifuged and the supernatant recovered, brought to a 0.2% (w/v) final concentration of Triton X-100, and dialyzed at 4°C. The cell extract was then precleared on 1 mg of uncoupled streptavidin-conjugated paramagnetic beads (Invitrogen Srl) on a rotating wheel, recovered and incubated with 1 mg of streptavidin-conjugated beads previously incubated with 2.5 nmoles of GroPIns-Bio or biotin in binding buffer (50 mM Tris-HCl, pH 7.6, 50 mM KCl, 10 mM EDTA) supplemented with the protease inhibitor cocktail. Following incubation, the unbound materials were separated and the beads were washed with binding buffer. GroPIns-bound proteins were specifically eluted with 5 mM GroPIns. The elution was performed for 30 min at 4°C on a rotating wheel, eluted proteins were recovered, resuspended in SDS sample buffer and analyzed by SDS-PAGE. Protein bands were analyzed by liquid chromatography coupled to tandem mass spectrometry (LC/MS-MS). For GroPIns-Bio pull-down assays with purified Bax, 100 ng of purified His-Bax were incubated for 2 h at 4°C with 0.5 mg of streptavidin-conjugated paramagnetic beads in the presence of 2.5 nmoles of biotin (Sigma-Aldrich, #B4501) or GroPIns-Bio in binding buffer plus protease inhibitors (Complete Mini EDTA-free, Roche). Following incubation, the unbound material was removed, and beads were washed with binding buffer. The beads with bound protein were boiled in 100 μl of SDS-sample buffer.

### Immunofluorescence and Confocal Microscopy

Cells (1**×**10^5^/sample) were cultured at 37°C in culture medium w/o BCS in the presence of 250 nM Mitotracker Orange (Invitrogen, Molecular Probes, #M7511) in the dark, then washed with PBS and treated for 20 min in culture medium w/o BCS at 37°C in the presence of 100 μM GroPIns, 35 μM Fludarabine or the combination of both. Diagnostic microscope slides were coated with polylysine (Sigma-Aldrich, #1274) and treated cells were allowed to adhere for 10 min. Slides were immediately fixed in methanol (Carlo Erba, #412383) at -20°C for 10 min as described (33). Following fixation, samples were washed 5 min in PBS and incubated with anti-Bax (B-9) primary antibodies o/n at 4°C or 1 h at RT. After washing in PBS, samples were incubated for 1 h at RT with Alexa Fluor 488-labeled secondary antibodies. Confocal microscopy was carried out on a Zeiss LSM700 using a 63× objective, as reported (33). Images were processed with Zen 2009 image software (Carl Zeiss, Jena, Germany) and analyses were performed using ImageJ software (downloaded from http://www.embl-heidelberg.de/eamnet/).

### RNA Isolation, Reverse Transcription and Real-Time Quantitative PCR

RNA was extracted and retrotranscribed as described (34). Real-time PCR was performed in triplicate on 96-well optical PCR plates (Sarstedt AG, Nümbrecht, Germany) using SSo FastTM EvaGreenR SuperMix and a CFX96 Real-Time system (Bio-Rad Laboratories, Waltham, MA). Results were processed and analyzed as described (34). Values are expressed as ΔΔCT relative to the housekeeping gene HPRT1. Primers used for real-time quantitative PCR amplification are listed in **Supplementary Table 2**.

### Statistical Analyses

One-way ANOVA with *post-hoc* Tukey was used for experiments where multiple groups were compared. Mann-Whitney rank-sum tests were performed to determine the significance of the differences between two groups. Statistical analyses were performed using GraphPad Software (La Jolla, CA). P values <0.05 were considered significant.

### Combination Index Calculation

The Combination index (Bliss index) was calculated according to the literature (35, 36). Briefly, CLL cells from 2 patients were mixed and plated into 96 well plates in 100 μl culture medium. GroPIns, Fludarabine and/or Venetoclax were added at different concentrations for 24 h, alone or in combination. Cell apoptosis was analyzed as above and the Combination index was calculated as in (35).

### Study Approval

Written informed consent was received from CLL patients and healthy donors prior to inclusion in the study according to the Declaration of Helsinki. Experiments were approved by the local Ethics Committee.

## Results

### GroPIns Has a Pro-Apoptotic Activity on CLL Cells Which Depends on SHP-1

**Figure 1:**
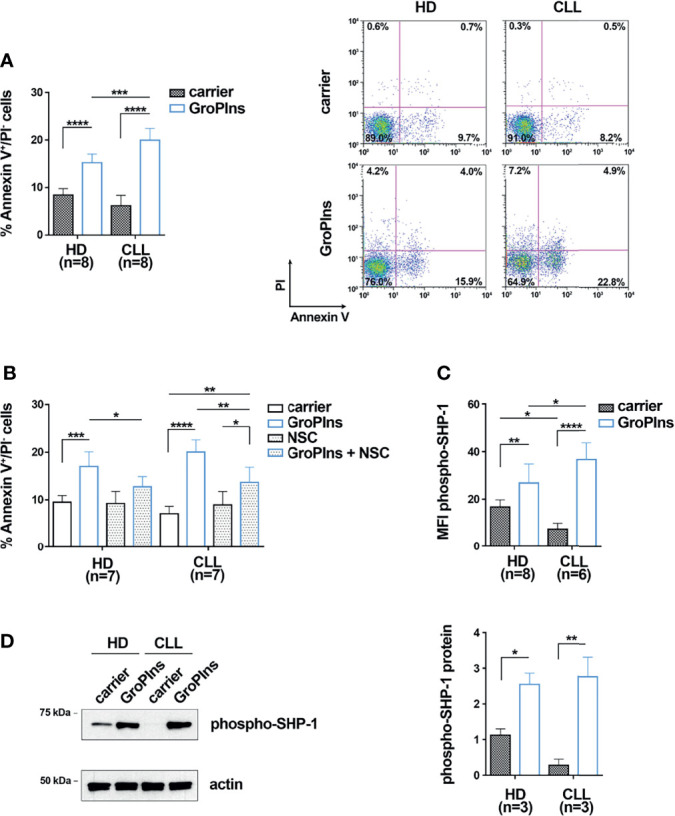
GroPIns promotes CLL cell apoptosis in a SHP-1-dependent manner. **(A)** Flow cytometric analysis of the percentages of Annexin V^+^/PI^-^ cells in B lymphocytes purified from peripheral blood of healthy donors (HD; n=8) and CLL patients (CLL; n=8). Samples were treated with either carrier or 100 μM GroPIns for 24 h at 37°C. Representative panels are shown on the right. **(B)** Flow cytometric analysis of the percentages of Annexin V^+^/PI^-^ cells in B lymphocytes purified from peripheral blood of healthy donors (HD; n=7) and CLL patients (CLL; n=7). Samples were treated for 24 h at 37°C with either carrier or 100 μM GroPIns in the presence or absence of 50 μM NSC-87887 (NSC). **(C)** Flow cytometric analysis of phospho-SHP-1 in B lymphocytes purified from peripheral blood of healthy donors (HD; n=8) and CLL patients (CLL; n=6), treated with either carrier or 100 μM GroPIns for 30 min at 37°C. Data are expressed as MFI phospho-SHP-1 in live cells. **(D)** Immunoblot analysis with anti-phospho-SHP-1 antibodies of postnuclear supernatants of B lymphocytes purified from peripheral blood of healthy donors (HD; n=3) and CLL patients (CLL; n=3). Samples were treated with either carrier or 100 μM GroPIns for 30 min at 37°C. The stripped filters were reprobed with anti-actin antibodies. Molecular weights (kDa) are indicated on the left of the panel. The quantification of three independent experiments is shown on the right. Mean ± SD. Anova two-way test, Multiple Comparison. p ≤ 0.0001, ****; p ≤ 0.001, ***; p ≤ 0.01, **; p ≤ 0.05, *.

The activity of the tyrosine phosphatase SHP-1, known to promote apoptosis (18, 25), has been shown to be impaired in CLL cells (26). Since GroPIns is a well-known regulator of SHP-1 in melanoma cells (17), we asked whether it promotes apoptosis of CLL cells through a SHP-1-dependent mechanism. B cells purified from peripheral blood of CLL patients were cultured for 24 h in the presence of 100 μM GroPIns and the percentage of early apoptotic Annexin V^+^/PI^-^ cells was quantified by flow cytometry. B cells from healthy donors were used as control. GroPIns enhanced apoptosis of CLL cells (**Figures 1A, B**; **Supplementary Figure 2**). Apoptosis of healthy B cells was also enhanced by GroPIns, although at significantly lower levels compared to CLL cells (**Figure 1A**). The pro-apoptotic activity of GroPIns was partly reversed by the SHP-1-specific inhibitor NSC-87887 (**Figure 1B**), demonstrating that the pro-apoptotic activity of GroPIns relies on the tyrosine phosphatase activity of SHP-1.

The active form of SHP-1 is phosphorylated on tyrosine 564 (37). We hypothesized that, similar to melanoma cells (17), GroPIns interacts with and activates SHP-1 in CLL cells, thereby promoting their apoptosis. To test this hypothesis, B cells purified from peripheral blood of CLL patients and healthy controls were cultured in the presence of GroPIns and the active, phosphorylated form of SHP-1 was quantified by flow cytometry using a phospho-Y564-specific antibody (37). Consistent with previous reports (26), basal SHP-1 phosphorylation levels were significantly lower in CLL cells compared to healthy B cells (**Figures 1C, D**; **Supplementary Figure 3**). GroPIns enhanced SHP-1 phosphorylation (**Figures 1C, D**). These data suggest that GroPIns promotes CLL cell apoptosis by activating SHP-1. However, the fact that the enhancing effects of GroPIns on B cell apoptosis were only partially reversed by the SHP-1 inhibitor suggests that other, SHP-1-independent mechanisms may contribute to this function.

### GroPIns Enhances the Expression of Bax in CLL Cells in a SHP-1-Dependent Manner

**Figure 2:**
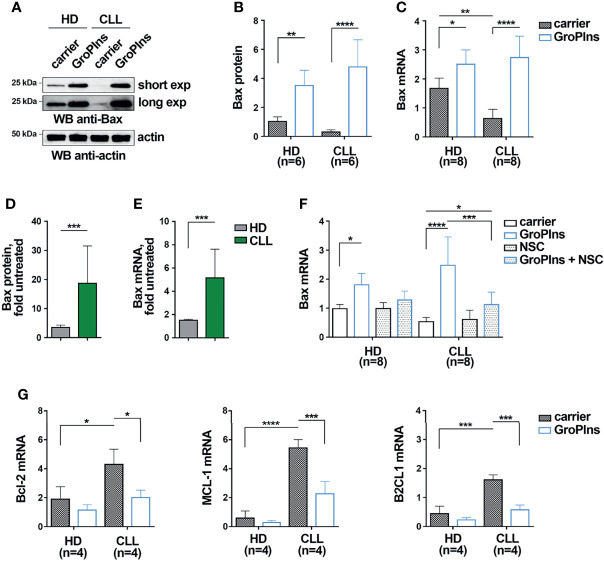
GroPIns promotes Bax expression in CLL cells. **(A, B)** Immunoblot analysis with anti-Bax antibodies of postnuclear supernatants of B lymphocytes purified from peripheral blood of healthy donors (HD; n=6) and CLL patients (CLL; n=6). Samples were treated with either carrier or 100 μM GroPIns for 24 h at 37°C. The stripped filters were reprobed with anti-actin antibodies. Molecular weights (kDa) are indicated on the left of the panel. The quantification of eight independent experiments is shown in **(B, C)**. Quantitative RT-PCR analysis of Bax mRNA in B lymphocytes purified from peripheral blood of healthy donors (HD; n=8) and CLL patients (CLL; n=8), treated with either carrier or 100 μM GroPIns for 24 h at 37°C. The relative gene transcript abundance was determined on triplicate samples using the ddCt method and normalized to HPRT1. **(D, E)**). Fold protein **(D)** and mRNA **(E)** expression levels of Bax in samples from healthy donors and CLL patients. Data were calculated as fold Bax protein quantification of treated *vs* untreated samples shown in **(B, C)**. **(F)** Quantitative RT-PCR analysis of Bax mRNA in B lymphocytes purified from peripheral blood of healthy donors (HD; n=8) and CLL patients (CLL; n=7), treated for 24 h at 37°C with either carrier or 100 μM GroPIns in the presence or absence of 50 μM NSC-87887 (NSC). The relative gene transcript abundance was determined on triplicate samples using the ddCt method and normalized to HPRT1. **(G)** Quantitative RT-PCR analysis of Bcl-2, MCL-1 and B2CL1 mRNA in B lymphocytes purified from peripheral blood of healthy donors (HD; n=4) and CLL patients (CLL; n=4), treated with either carrier or 100 μM GroPIns for 24 h at 37°C. The relative gene transcript abundance was determined on triplicate samples using the ddCt method and normalized to HPRT1. Mean ± SD. **(B, C, F, G)**: Anova two-way test, Multiple Comparison. **(D, E)**: Mann Whitney Rank Sum Test. p ≤ 0.0001, ****; p ≤ 0.001, ***; p ≤ 0.01, **; p ≤ 0.05, *.

The apoptosis defects of CLL cells are caused in part by the decreased expression of the pro-apoptotic protein Bax (2). Since the phosphatase activity of SHP-1 has been causally linked to enhanced Bax expression and increased apoptosis in acute promyelocytic leukemia cells (25), we asked whether GroPIns promotes CLL cell apoptosis by upregulating Bax expression in a SHP-1-dependent manner. B cells purified from peripheral blood of CLL patients and healthy donors were cultured for 24 h in the presence of GroPIns. Bax expression was assessed by both immunoblot and qRT-PCR. Consistent with previous reports (2, 5), untreated CLL cells expressed lower Bax levels compared to healthy B cells (**Figures 2A–C**). GroPIns enhanced Bax expression in both CLL cells and healthy B cells (**Figures 2A–C**). Although the overall protein and mRNA amount of Bax was similar in healthy and CLL cells treated with GroPIns, the fold Bax expression, calculated as the ratio of Bax expression in treated versus untreated samples, was significantly higher in CLL cells compared to healthy B cells (**Figures 2D, E**). These results suggest a higher sensitivity of CLL cells to GroPIns compared to healthy B cells. NSC-87887 almost completely abolished the GroPIns-dependent Bax increase, demonstrating that the Bax-elevating activity of GroPIns depends on the phosphatase activity of SHP-1 (**Figure 2F**). Hence GroPIns promotes CLL cell apoptosis by enhancing Bax expression in a SHP-1-dependent manner. Of note, GroPIns also decreased the mRNA expression of the pro-survival Bcl-2 family members Bcl-2, MCL-1 and B2CL1 in CLL cells (**Figure 2G**) in a SHP-1-dependent manner (**Supplementary Figure 4**). These data provide evidence that GroPIns profoundly shifts the Bcl-2 family balance toward apoptosis.

### GroPIns Interacts With and Activates Bax in CLL Cells

**Figure 3:**
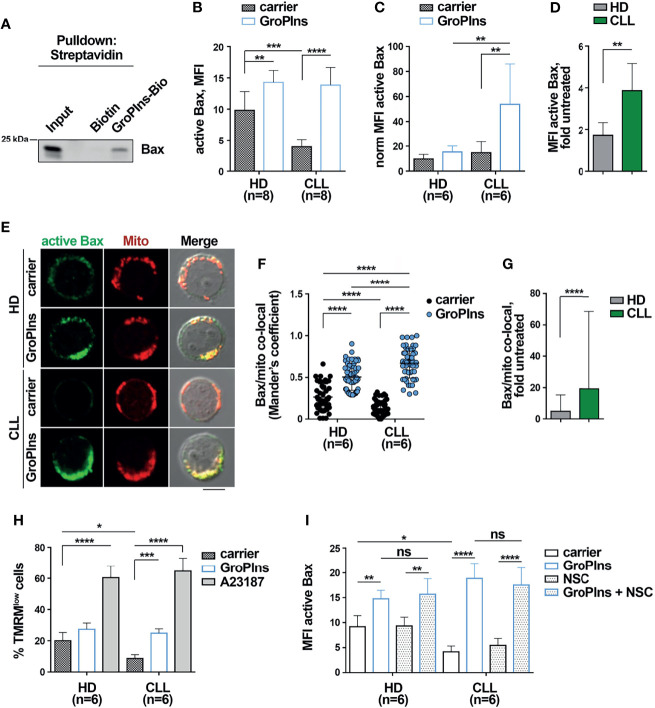
GroPIns interacts with and activates Bax. **(A)** Representative pull-down of streptavidin-conjugated beads using Biotin or biotinylated GroPIns (GroPIns-Bio) with His-Bax. Eluted proteins were analyzed by immunoblot using anti-His antibodies. Molecular weights (kDa) are indicated on the left of the panel. **(B)** Flow cytometric analysis of active Bax in B lymphocytes purified from peripheral blood of healthy donors (HD; n=8) and CLL patients (CLL; n=8). Samples were treated for 20 min at 37°C with either carrier or 100 μM GroPIns. **(C)** The MFI of active Bax shown in panel **(B)** was normalized to Bax protein levels of untreated cells shown in **Figure 2B** (n=6). **(D)** Fold MFI active Bax in samples from healthy donors and CLL patients shown in panel **(C)**. Data were calculated as fold MFI of active Bax of treated vs. untreated samples. **(E)** Immunofluorescence analysis of active Bax (green) and mitochondria (Mitotracker) (red) in B lymphocytes purified from peripheral blood of healthy donors (HD; n=6) and CLL patients (CLL; n=6) treated for 20 min at 37°C with either carrier or 100 μM GroPIns. Immunofluorescence images were acquired on confocal microscope using 60 × objective. Representative immunofluorescence images are shown. Size bar, 5 μm. The quantification using Mander’s coefficient of the weighted colocalization of active Bax with mitochondria in individual medial confocal sections is shown in **(F)**. **(G)** Fold active Bax/mitochondria co-localization in cells from healthy donors and CLL patients. Data were calculated as fold active Bax/mitochondria co-localization of treated *vs* untreated samples. **(H)** Flow cytometric analysis of the percentage of TMRM^low^ cells in B lymphocytes purified from peripheral blood of healthy donors (HD; n=6) and CLL patients (CLL; n=6). Samples were treated for 4 h at 37°C with either carrier or 100 μM GroPIns or 500 ng/ml A23187. Stainings were performed in duplicate. **(I)** Flow cytometric analysis of active Bax in B lymphocytes purified from peripheral blood of healthy donors (HD; n=6) and CLL patients (CLL; n=6). Samples were treated for 20 min at 37°C with either carrier or 100 μM GroPIns in the presence or in the absence of NSC-87887. Mean ± SD. **(B, C, F, H, I)**: Anova two-way test, Multiple Comparison. **(D, G)**: Mann Whitney Rank Sum Test. p ≤ 0.0001, ****; p ≤ 0.001, ***; p ≤ 0.01, **; p ≤ 0.05, *; ns, not significant..

We previously identified SHP-1 as a direct cellular target of GroPIns by pull-down assay coupled with liquid chromatography-tandem mass-spectrometry analysis (16). Among direct interactors of GroPIns (listed in **Table 1**) we also found Bax. We validated the direct binding of GroPIns with Bax in *in vitro* pull-down assays. The immunoblot analysis of Bax showed that purified recombinant Bax was specifically pulled-down by GroPIns-Bio-bound beads but not by control Biotin-bound beads, confirming that GroPIns directly binds Bax (**Figure 3A**).

**Table 1:** List of proteins identified from proteomic analysis.

Swiss-Prot Code	Protein name
O55143	Sarcoplasmic/endoplasmic reticulum calcium ATPase 2
Q8CGC7	Bifunctional glutamate/proline–tRNA ligase
Q9JKR6	Hypoxia up-regulated protein 1
Q8BMJ2	Leucine–tRNA ligase, cytoplasmic
P70248	Unconventional myosin-If
Q64514	Tripeptidyl-peptidase 2
Q8K4Z5	Splicing factor 3A subunit 1
Q9EQK5	Major vault protein
Q60597	2-oxoglutarate dehydrogenase, mitochondrial
Q8BIJ6	Isoleucine–tRNA ligase, mitochondrial
Q9DBT5	AMP deaminase 2
Q61881	DNA replication licensing factor MCM7
Q9D0R2	Threonine–tRNA ligase 1, cytoplasmic
Q9JIK5	Nucleolar RNA helicase 2
Q9Z110	Delta-1-pyrroline-5-carboxylate synthetase
P26043	Radixin
Q80UM7	Mannosyl-oligosaccharide glucosidase
Q8BML9	Glutamine–tRNA ligase
Q8CHW4	Translation initiation factor eIF-2B subunit epsilon
Q8BNW9	Kelch repeat and BTB domain-containing protein 11
Q99MN1	Lysine–tRNA ligase
Q9WUA2	Phenylalanine–tRNA ligase beta subunit
P29351	Tyrosine-protein phosphatase non-receptor type 6 (Shp1)
P80316	T-complex protein 1 subunit epsilon
Q8BMF4	Dihydrolipoamide acetyltransferase PDH-E2
Q8BP47	Asparagine–tRNA ligase, cytoplasmic
Q91WQ3	Tyrosine–tRNA ligase, cytoplasmic
Q9DBG6	Dolichyl-diphosphooligosaccharide–protein glycosyltransferase subunit 2
Q61024	Asparagine synthetase
P09405	Nucleolin
Q61656	Probable ATP-dependent RNA helicase DDX5
P30416	Peptidyl-prolyl cis-trans isomerase FKBP4
Q99K87	Serine hydroxymethyltransferase, mitochondrial
P47738	Aldehyde dehydrogenase, mitochondrial
Q9Z0N1	Eukaryotic translation initiation factor 2 subunit 3
P80314	T-complex protein 1 subunit beta
P26443	Glutamate dehydrogenase 1, mitochondrial
Q9CZ44	NSFL1 cofactor p47
O88986	2-amino-3-ketobutyrate coenzyme A ligase, mitochondrial
Q922R8	Protein disulfide-isomerase A6
Q9DC69	NADH dehydrogenase 1 alpha subcomplex subunit 9
Q9DB05	Alpha-soluble NSF attachment protein
Q99LC5	Electron transfer flavoprotein subunit alpha, mitochondrial
Q64674	Spermidine synthase
Q9CR57	60S ribosomal protein L14
P35278	Ras-related protein Rab-5C
P84099	60S ribosomal protein L19
P20108	Thioredoxin-dependent peroxide reductase, mitochondrial
P61087	Ubiquitin-conjugating enzyme E2 K
P08030	Adenine phosphoribosyltransferase
P62821	Ras-related protein Rab-1A
Q9CZM2	60S ribosomal protein L15
Q9Z1B5	Mitotic spindle assembly checkpoint protein MAD2A
Q62159	Rho-related GTP-binding protein RhoC
P51410	60S ribosomal protein L9
Q9JM14	5’(3’)-deoxyribonucleotidase, cytosolic type
P61028	Ras-related protein Rab-8B
P29391	Ferritin light chain 1
P53994	Ras-related protein Rab-2A
P70296	Phosphatidylethanolamine-binding protein 1
P19253	60S ribosomal protein L13a
P08030	Adenine phosphoribosyltransferase
P00375	Dihydrofolate reductase
O09167	60S ribosomal protein L21
Q07813	Apoptosis regulator BAX
Q9EQU5	Protein SET
P62301	40S ribosomal protein S13
P17742	Peptidyl-prolyl cis-trans isomerase A
P62281	40S ribosomal protein S11

Following pro-apoptotic stimulation, Bax undergoes a conformational change to become an active apoptosis promoter (9, 11). We assessed whether GroPIns promotes Bax activation. Purified healthy and CLL cells were treated with GroPIns for 20 min and Bax activation was assessed by flow cytometric analysis of cells stained with an anti-active Bax antibody that specifically recognizes the N-terminus of Bax which is exposed after the conformational change that accompanies Bax activation (9). The basal levels of Bax activation were significantly lower in CLL cells compared to healthy B cells (**Figures 3B, D**; **Supplementary Figure 3**). This was a consequence of the lower overall Bax levels, as assessed by normalizing the MFI of active Bax to the expression levels of Bax protein shown in **Figure 2B** (**Figure 3C**). GroPIns elicited Bax activation in CLL cells (**Figures 3B, C**). The fold Bax activation, calculated as the ratio of the MFI of active Bax in treated versus untreated samples, was significantly higher in CLL cells compared to healthy B cells (**Figure 3D**), further witnessing to a higher sensitivity of CLL cells to GroPIns compared to healthy B cells.

Active Bax translocates to the mitochondria (11). Immunofluorescence analysis of cells stained with anti-active Bax antibodies and Mitotracker Orange, a fluorescent probe that selectively stains mitochondria, showed that the colocalization of active Bax with mitochondria was significantly enhanced in both healthy and CLL cells treated for 20 min with GroPIns compared to untreated cells (**Figures 3E, F**). The fold active Bax/mitochondria co-localization was significantly higher in CLL cells compared to healthy B cells (**Figure 3G**), again demonstrating the higher sensitivity of leukemic cells to GroPIns.

Bax translocation to mitochondria leads to its oligomerization at the outer mitochondrial membrane, which in turn promotes mitochondrial depolarization (11). Purified healthy and CLL cells loaded with the fluorescent probe TMRM were treated for 4 h with GroPIns or with the calcium ionophore A23187, a potent inducer of apoptosis (38), and mitochondria depolarization was assessed by flow cytometric quantification of the percentage of TMRM^low^ cells (**Supplementary Figure 5**). Mitochondrial depolarization was significantly enhanced in CLL cells treated with GroPIns when compared to untreated cells (**Figure 3H**). Of note, GroPIns elicited a slight, yet not significant increase in mitochondrial depolarization in healthy B cells (**Figure 3H**). These data demonstrate that GroPIns potently acts on CLL cells to restore apoptosis. The SHP-1 inhibitor NSC-87887 did not impair GroPIns-mediated Bax activation (**Figure 3I**), suggesting that GroPIns-mediated Bax activation does not require SHP-1. Collectively, these results support the existence of two unrelated pathways, of which one is SHP-1-dependent and one independent, converging on Bax and exploited by GroPIns to promote CLL cell apoptosis.

### GroPIns Enhances the Pro-Apoptotic Effects of Venetoclax on CLL Cells

**Figure 4:**
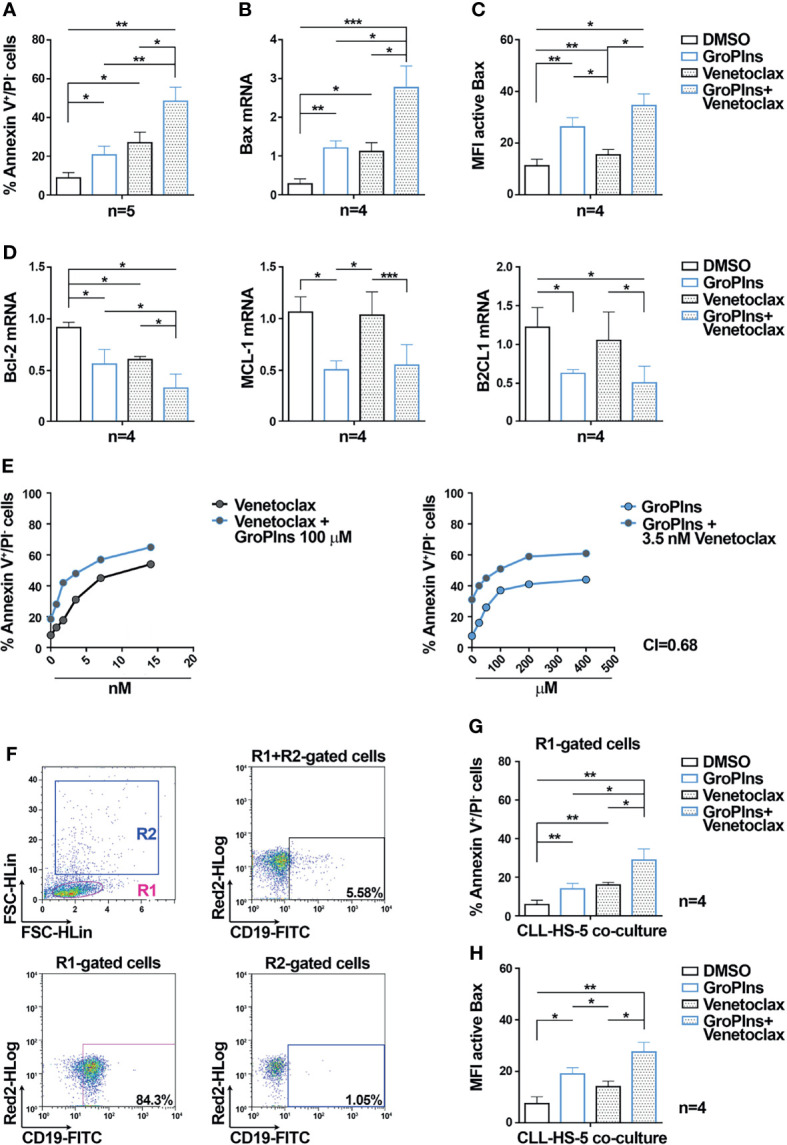
GroPIns enhances the pro-apoptotic activity of Venetoclax in CLL cells. **(A)** Flow cytometric analysis of the percentages of Annexin V^+^/PI^-^ cells in B lymphocytes purified from peripheral blood of CLL patients (CLL; n=5) treated with either 100 μM GroPIns or 3.5 nM Venetoclax or the combination of both for 24 h at 37°C. **(B)** Quantitative RT-PCR analysis of Bax mRNA in B lymphocytes purified from peripheral blood of CLL patients (CLL; n=4) and treated as in **(A)**. The relative gene transcript abundance was determined on triplicate samples using the ddCt method and normalized to HPRT1. **(C)** Flow cytometric analysis of active Bax in B lymphocytes purified from peripheral blood of CLL patients (CLL; n=4) and treated for 20 min at 37°C with either 100 μM GroPIns or 3.5 nM Venetoclax or the combination of both. **(D)** Quantitative RT-PCR analysis of Bcl-2, MCL-1 and B2CL1 mRNA in B lymphocytes purified from peripheral blood of healthy donors (HD; n=4) and CLL patients (CLL; n=4), treated as above. The relative gene transcript abundance was determined on triplicate samples using the ddCt method and normalized to HPRT1. **(E)** Flow cytometric analysis of the percentages of Annexin V^+^/PI^-^ cells in B lymphocytes purified from peripheral blood of 2 CLL patients treated with either GroPIns or Venetoclax or with the combination of both at the indicated concentrations for 24 h at 37°C. The calculated Cooperation Index (CI) is indicated. **(F–H)** Flow cytometric analysis of the percentages of Annexin V^+^/PI^-^ cells **(G)** and of Bax activation **(H)** in B lymphocytes purified from peripheral blood of CLL patients (CLL; n=4) co-cultured with HS-5 stromal cells for 24 h at 37°C in the presence of either 100 μM GroPIns or 3.5 nM Venetoclax or the combination of both. Analysis was carried out on R1-gated CD19^+^ cells. The gating strategy is shown in **(F)**. Mean ± SD. Anova one-way test, Multiple Comparison. p ≤ 0.001, ***; p ≤ 0.01, **; p ≤ 0.05, *.

The Bcl-2 inhibitor Venetoclax promotes CLL cell apoptosis (39), and induces rapid and pronounced activation and mitochondrial translocation of Bax in cell lines of acute myeloid leukemia (40). We tested whether the combination of GroPIns with Venetoclax further enhances Venetoclax-induced CLL cell apoptosis. As shown in **Figure 4A**, the combination of GroPIns and Venetoclax enhanced apoptosis of leukemic cells compared to single treatments (**Figure 4A**), suggesting a synergic pro-apoptotic activity of GroPIns and Venetoclax in these cells. This was confirmed by analyzing Bax expression (**Figure 4B**) and activation (**Figure 4C**), which were enhanced in CLL cells subjected to combination treatments compared to single treatments (**Figures 4B, C**). Of note, while Venetoclax did not affect the expression of MCL-1 and B2CL1 in CLL cells, it led to a decrease in Bcl-2 expression to levels similar to GroPIns, which were further decreased in combination treatments (**Figure 4D**). The flow cytometric analysis of early apoptotic cells performed in CLL cells treated for 24 h with increasing concentrations of GroPIns alone or in combination with Venetoclax showed a Combination Index (CI) below 1 (CI=0.68; **Figure 4E**), indicating a synergic cooperation between GroPIns and Venetoclax to promote CLL cell apoptosis.

Fludarabine, a chemotherapeutic drug used in the treatment of a small subset of CLL patients alone or in combination with other chemotherapeutic or immunomodulatory drugs, enhances Bax activation and expression and promotes apoptosis of CLL cells (9, 10). We tested whether, similar to Venetoclax, the combination of GroPIns with Fludarabine further enhances Fludarabine-induced CLL cell apoptosis. GroPIns enhanced Fludarabine-induced CLL cell apoptosis as well as Bax activation, expression and translocation to mitochondria compared to single treatments (**Supplementary Figure 6A-F**). However, as opposed to Venetoclax, Fludarabine and GroPIns did not act in synergy to enhance CLL cell apoptosis, but rather showed independent effects (**Supplementary Figure 6G**).

The stromal microenvironment strongly contributes to protect CLL cells from apoptosis (6). We assessed the pro-apoptotic effect of GroPIns and Venetoclax, alone or in combination treatments, in CLL cells co-cultured for 24 h with the human stromal cell line HS-5 (29). As shown in **Figures 4F–H**, the combination of GroPIns and Venetoclax enhanced both apoptosis and Bax activation in leukemic cells co-cultured with HS-5 cells compared to single treatments (**Figure 4A**), albeit with less pronounced effects which are likely to be accounted for by the protective role of stromal cells on CLL cells.

These results demonstrate that GroPIns displays a pro-apoptotic activity also in the presence of drugs known to promote CLL cell apoptosis.

## Discussion

Apoptosis, which plays important roles in organism development and tissue homeostasis, becomes critical for the elimination of unwanted, damaged or infected cells (41). Insufficient apoptosis has been related to the onset and progression of cancer by extending tumor cell survival and promoting their resistance to treatment (42). A profound imbalance among Bcl-2 family members is a major factor in the apoptosis defects of CLL cells, which play a major role in leukemic cell accumulation in secondary lymphoid organs, where they are protected from chemotherapy (1, 2). The pro-survival protein Bcl-2, whose expression is frequently upregulated in CLL as a result of deletion of mir15-a/mir16-1, located at 13q14 and known to target BCL-2 mRNA (43), had long been viewed as a promising target for CLL therapy. In 2016 the selective Bcl-2 inhibitor Venetoclax, which acts as a BH3-mimetic to facilitate the activation of pro-apoptotic Bcl-2 family members, was approved for relapsed/refractory CLL (7). Since then, new combination therapy regimens have been approved for CLL treatment (8) and usually applied as first-line therapy. The use of chemoimmunotherapeutics such as Fludarabine, cyclophosphamide and rituximab has progressively decreased through the years as a consequence of the higher efficacy and better tolerability of targeted agents like Venetoclax. However, none of the recently introduced therapies appears to cure CLL, and some patients become resistant to Venetoclax due to the acquisition of Bcl-2 mutations.

Pro-apoptotic stimuli activate Bax, a major pro-apoptotic member of the Bcl-2 family, either directly or indirectly, leading to mitochondrial membrane permeabilization, release of the apoptotic factor cytochrome c and cancer cell death (11). The expression of Bax is profoundly impaired in CLL cells (2), which contributes to their apoptosis defects. A number of drugs currently in clinical use for the treatment of several types of cancer are known to indirectly enhance Bax expression and activation, including Fludarabine (9, 10) and Venetoclax (40). Here we demonstrate that GroPIns promotes CLL cell apoptosis by enhancing Bax expression. Moreover, GroPIns enhances the pro-apoptotic effects of both Venetoclax and Fludarabine, leading to higher levels of CLL cell apoptosis compared to single treatments. Interestingly, the activity of GroPIns and Venetoclax converge toward tilting the Bcl-2 family balance toward apoptosis, on the one hand by enhancing the expression and potentiating the activation of Bax, and on the other hand by decreasing the expression and inhibiting the activity of Bcl-2. Our findings highlight a potential new combinatorial strategy aimed at potentiating the pro-apoptotic activity of Venetoclax with a natural and well-tolerated compound, which could overcome potential resistance mechanisms to Venetoclax used as single agent (44).Several classes of small molecules have been identified in the last decade that selectively activate Bax to induce apoptosis, which demonstrated good *in vitro* but moderate *in vivo* anti-cancer activity (45, 46). The compound SMBA1 potently activates Bax and acts both *in vitro* and *in vivo* against lung cancer (47). New recently synthesized SMBA1 analogs show anti-proliferative activity against breast cancer (48). However, none of these molecules has been tested in CLL to date. In 2020 the small molecule BDA-366, a BH4-domain antagonist that kills both lung cancer and multiple myeloma cells, was tested for its therapeutic potential and mechanism of action in CLL and DLBCL. However, although BDA-366 displayed selective toxicity against both cell types, the underlying mechanism of Bax activation is as yet unknown (11, 49). Here we identified GroPIns as a naturally-occurring molecule provided with the intrinsic ability to bind and activate Bax. This makes of GroPIns an interesting pro-apoptotic molecule to be tested in malignancies characterized by hypoexpression or hypoactivation of Bax.

Along with an aberrant expression of anti-apoptotic molecules, CLL cells show high levels of intracellular phosphorylation mediated by the hyperactivation of several kinases downstream of the B-cell receptor, such as Lyn, Syk, Btk, PI3K, and AKT (50, 51). This condition is further sustained by an impairment in the expression or function of phosphatases. The expression of PTEN (52), CD45 (53), PTPROt (54), PHLPP1 (55, 56), PP2A (57), and SHIP1 (58) are significantly decreased in CLL cells, whereas PTPN22, which acts as a positive regulator of anti-apoptotic signals by hampering the negative regulation of B-cell receptor-dependent signaling pathways, is overexpressed (59). By contrast SHP-1, a tyrosine phosphatase that participates in signaling pathways regulating proliferation, survival and apoptosis of both hematopoietic and non-hematopoietic cells (18), is expressed in CLL cells at levels comparable to normal B cells (60) but is functionally dysregulated by mechanisms that are mediated by the Src family kinase Lyn (26), making this phosphatase an interesting target for activating-drug discovery.

Drugs able to promote phosphatase activity have been demonstrated to be effective in CLL. The novel SHIP-1 activator AQX-435 was demonstrated to be effective in the inhibition of anti-IgM-induced AKT phosphorylation, resulting in CLL cell apoptosis *in vitro* (61). Conversely, SHP-1 has proven to be an extremely challenging drug target, due both to the highly conserved and positively charged nature of its phosphatase active site, and to the lack of either appropriate selectivity or membrane permeability of the majority of phosphatase inhibitors (62). We previously reported that in melanoma cells GroPIns interacts with SHP-1, promoting its recruitment to invadopodia where it dephosphorylates critical components of the actin polymerization pathways leading to matrix invasion, thereby counteracting metastasis (17). Here we added a tile to the puzzle by demonstrating that in CLL cells GroPIns enhances SHP-1 phosphorylation. Although the molecular mechanism underlying the GroPIns-dependent enhancement in SHP-1 phosphorylation remains unknown, we hypothesize that the interaction of GroPIns with SHP-1 might either stabilize SHP-1 in an active conformation, or alternatively promote its interaction with a specific kinase, thereby favoring SHP-1 phosphorylation. It is noteworthy that SHP-1 not only acts through dephosphorylation (18), but also promotes Bax expression (23, 25) through signaling pathways involving the MAP kinase p38 (25) and the transcription factor STAT3 (23). Our data show that, by promoting SHP-1 phosphorylation, GroPIns enhances Bax expression and CLL cell apoptosis. The existence of two distinct and independent pathways that, by taking advantage of the two GroPIns interactors SHP-1 and Bax, both converge to promote CLL cell apoptosis, contribute to enhance the activity of this compound. In this scenario GroPIns, *via* direct binding to and modulation of SHP-1 and Bax, could be an interesting tool to restore apoptosis in CLL cells.

## Data Availability Statement

The original contributions presented in the study are included in the article/**Supplementary Material**. Further inquiries can be directed to the corresponding authors.

## Ethics Statement

The studies involving human participants were reviewed and approved by Azienda Ospedaliera Universitaria di Padova, Padova Hospital and Azienda Ospedaliera Universitaria di Siena, Siena Hospital. The patients/participants provided their written informed consent to participate in this study.

## Author Contributions

GB, ASV, VT, NC, FF, ADV, LT, DC, LP, and CB designed research and analyzed and interpreted data. GB, ASV, VT, NC, FF, ADV, and LP performed research. ASV, FF, ADV, LT, and DC contributed vital reagents. ASV, NC, FF, ADV, LT, DC, LP, and CB drafted the manuscript. All authors contributed to the article and approved the submitted version.

## Funding

The research leading to these results has received funding from AIRC under IG 2017 - ID. 20148 – and Regione Toscana, ID. PRECISE-CLL- to Baldari Cosima. This work was also supported by grants from AIRC to LT (IG-25024) and AIRC (IG-18776), PRIN (20177XJCHX) and the SATIN project 2014–2020 to DC.

## Conflict of Interest

The authors declare that the research was conducted in the absence of any commercial or financial relationships that could be construed as a potential conflict of interest.

## Publisher’s Note

All claims expressed in this article are solely those of the authors and do not necessarily represent those of their affiliated organizations, or those of the publisher, the editors and the reviewers. Any product that may be evaluated in this article, or claim that may be made by its manufacturer, is not guaranteed or endorsed by the publisher.

## References

[1] Kipps TJ Stevenson FK Wu CJ Croce CM Packham G Wierda WG. Chronic Lymphocytic Leukaemia. Nat Rev Dis Prim (2017) 3:16096. doi: 10.1038/nrdp.2016.96 PMC5336551 28102226

[2] Packham G Stevenson FK. Bodyguards and Assassins: Bcl-2 Family Proteins and Apoptosis Control in Chronic Lymphocytic Leukaemia. Immunology (2005) 114:441–9. doi: 10.1111/j.1365-2567.2005.02117.x PMC1782118 15804279

[3] Pekarsky Y Croce CM. Role of miR-15/16 in CLL. Cell Death Differ (2015) 22:6–11. doi: 10.1038/cdd.2014.87 24971479 PMC4262785

[4] Saxena A Viswanathan S Moshynska O Tandon P Sankaran K Sheridan DP. Mcl-1 and Bcl-2/Bax Ratio Are Associated With Treatment Response But Not With Rai Stage in B-Cell Chronic Lymphocytic Leukemia. Am J Hematol (2004) 75:22–33. doi: 10.1002/ajh.10453 14695629

[5] Capitani N Lucherini OM Sozzi E Ferro M Giommoni N Finetti F. Impaired Expression of p66Shc, a Novel Regulator of B-Cell Survival, in Chronic Lymphocytic Leukemia. Blood (2010) 115:3726–36. doi: 10.1182/blood-2009-08-239244 20061561

[6] Ten Hacken E Burger JA. Microenvironment Interactions and B-Cell Receptor Signaling in Chronic Lymphocytic Leukemia: Implications for Disease Pathogenesis and Treatment. Biochim Biophys Acta (2016) 1863:401–13. doi: 10.1016/j.bbamcr.2015.07.009 PMC4715999 26193078

[7] Roberts AW Davids MS Pagel JM Kahl BS Puvvada SD Gerecitano JF. Targeting BCL2 With Venetoclax in Relapsed Chronic LymphocyticLeukemia. N Engl J Med (2016) 374:311. doi: 10.1056/NEJMOA1513257 26639348 PMC7107002

[8] Hallek M. Chronic Lymphocytic Leukemia: 2020 Update on Diagnosis, Risk Stratification and Treatment. Am J Hematol (2019) 94:1266–87. doi: 10.1002/AJH.25595 31364186

[9] Bellosillo B Villamor N López-Guillermo A Marcé S Bosch F Campo E. Spontaneous and Drug-Induced Apoptosis Is Mediated by Conformational Changes of Bax and Bak in B-Cell Chronic Lymphocytic Leukemia. Blood (2002) 100:1810–6. doi: 10.1182/blood-2001-12-0327 12176904

[10] Zhou Z Fang Q Li P Ma D Zhe N Ren M. Entinostat Combined With Fludarabine Synergistically Enhances the Induction of Apoptosis in TP53 Mutated CLL Cells *via* the HDAC1/HO-1 Pathway. Life Sci (2019) 232:116583. doi: 10.1016/j.lfs.2019.116583 31226417

[11] Liu Z Ding Y Ye N Wild C Chen H Zhou J. Direct Activation of Bax Protein for Cancer Therapy. Med Res Rev (2016) 36:313–41. doi: 10.1002/med.21379 PMC4752390 26395559

[12] Valitutti S Cucchi P Colletta G Di Filippo C Corda D. Transformation by the K-Ras Oncogene Correlates With Increases in Phospholipase A2 Activity, Glycerophosphoinositol Production and Phosphoinositide Synthesis in Thyroid Cells. Cell Signal (1991) 3:321–32. doi: 10.1016/0898-6568(91)90061-X 1657098

[13] Corda D Zizza P Varone A Filippi BM Mariggiò S. The Glycerophosphoinositols: Cellular Metabolism and Biological Functions. Cell Mol Life Sci (2009) 66:3449–67. doi: 10.1007/s00018-009-0113-4 PMC11115907 19669618

[14] Patrussi L Mariggiò S Corda D Baldari CT. The Glycerophosphoinositols: From Lipid Metabolites to Modulators of T-Cell Signaling. Front Immunol (2013) 4:213. doi: 10.3389/fimmu.2013.00213 23908653 PMC3725514

[15] Vessichelli M Mariggiò S Varone A Zizza P Di Santo A Amore C. The Natural Phosphoinositide Derivative Glycerophosphoinositol Inhibits the Lipopolysaccharide-Induced Inflammatory and Thrombotic Responses. J Biol Chem (2017) 292:12828–41. doi: 10.1074/jbc.M116.773861 PMC5546025 28600357

[16] Varone A Mariggiò S Patheja M Maione V Varriale A Vessichelli M. A Signalling Cascade Involving Receptor-Activated Phospholipase A2, Glycerophosphoinositol 4-Phosphate, Shp1 and Src in the Activation of Cell Motility. Cell Commun Signal (2019) 17:20. doi: 10.1186/s12964-019-0329-3 PMC6396489 30823936

[17] Varone A Amoruso C Monti M Patheja M Greco A Auletta L. The Phosphatase Shp1 Interacts With and Dephosphorylates Cortactin to Inhibit Invadopodia Function. Cell Commun Signal (2021) 19:64. doi: 10.1186/s12964-021-00747-6 PMC8176763 34088320

[18] Varone A Spano D Corda D. Shp1 in Solid Cancers and Their Therapy. Front Oncol (2020) 10:935. doi: 10.3389/fonc.2020.00935 32596156 PMC7300250

[19] Zhang Q Wang HY Marzec M Raghunath PN Nagasawa T Wasik MA. STAT3- and DNA Methyltransferase 1-Mediated Epigenetic Silencing of SHP-1 Tyrosine Phosphatase Tumor Suppressor Gene in Malignant T Lymphocytes. Proc Natl Acad Sci USA (2005) 102:6948–53. doi: 10.1073/pnas.0501959102 PMC1100783 15870198

[20] Wen LZ Ding K Wang ZR Ding CH Lei SJ Liu JP. SHP-1 Acts as a Tumor Suppressor in Hepatocarcinogenesis and HCC Progression. Cancer Res (2018) 78:4680–91. doi: 10.1158/0008-5472.CAN-17-3896 29776962

[21] Koh JS Joo MK Park JJ Yoo HS Il CB BJ L. Inhibition of STAT3 in Gastric Cancer: Role of Pantoprazole as SHP-1 Inducer. Cell Biosci (2018) 8:50. doi: 10.1186/s13578-018-0248-9 PMC6127946 30202514

[22] Sharma Y Ahmad A Bashir S Elahi A Khan F. Implication of Protein Tyrosine Phosphatase SHP-1 in Cancer-Related Signaling Pathways. Futur Oncol (2016) 12:1287–98. doi: 10.2217/fon-2015-0057 26987952

[23] Chen H Zhu B Zhao L Liu Y Zhao F Feng J. Allicin Inhibits Proliferation and Invasion *In Vitro* and *In Vivo via* SHP-1-Mediated STAT3 Signaling in Cholangiocarcinoma. Cell Physiol Biochem (2018) 47:641–53. doi: 10.1159/000490019 29794468

[24] Saraswati S Alhaider A Abdelgadir AM Tanwer P Korashy HM. Phloretin Attenuates STAT-3 Activity and Overcomes Sorafenib Resistance Targeting SHP-1–Mediated Inhibition of STAT3 and Akt/VEGFR2 Pathway in Hepatocellular Carcinoma. Cell Commun Signal (2019) 17:127. doi: 10.1186/S12964-019-0430-7 PMC6794763 31619257

[25] Gan L Zhong L Shan Z Xiao C Xu T Song H. Epigallocatechin-3-Gallate Induces Apoptosis in Acute Promyelocytic Leukemia Cells *via* a SHP-1-P38α MAPK-Bax Cascade. Oncol Lett (2017) 14:6314–20. doi: 10.3892/ol.2017.6980 PMC5661390 29113283

[26] Tibaldi E Pagano MA Frezzato F Trimarco V Facco M Zagotto G. Targeted Activation of the SHP-1/PP2A Signaling Axis Elicits Apoptosis of Chronic Lymphocytic Leukemia Cells. Haematologica (2017) 102:1401–12. doi: 10.3324/haematol.2016.155747 PMC5541874 28619847

[27] Hallek M Cheson BD Catovsky D Caligaris-Cappio F Dighiero G Döhner H. Hallek 2008 Blood Glines for Diagnois and Treament of Chrn Lymph Leuk. Blood (2008) 111:5446–56. doi: 10.1182/blood-2007-06-093906 PMC2972576 18216293

[28] Visentin A Facco M Gurrieri C Pagnin E Martini V Imbergamo S. Prognostic and Predictive Effect of IGHV Mutational Status and Load in Chronic Lymphocytic Leukemia: Focus on FCR and BR Treatments. Clin Lymphoma Myeloma Leuk (2019) 19:678–685.e4. doi: 10.1016/j.clml.2019.03.002 31371221

[29] Patrussi L Manganaro N Capitani N Ulivieri C Tatangelo V Libonati F. Enhanced IL-9 Secretion by p66Shc-Deficient CLL Cells Modulates the Chemokine Landscape of the Stromal Microenvironment. Blood (2021) 137:2182–95. doi: 10.1182/blood.2020005785 33181836

[30] Roecklein BA Torok-Storb B. Functionally Distinct Human Marrow Stromal Cell Lines Immortalized by Transduction With the Human Papilloma Virus E6/E7 Genes. Blood (1995) 85:997–1005. doi: 10.1182/blood.V85.4.997.bloodjournal854997 7849321

[31] Patrussi L Capitani N Cannizzaro E Finetti F Lucherini OM Pelicci PG. Negative Regulation of Chemokine Receptor Signaling and B-Cell Chemotaxis by p66Shc. Cell Death Dis (2014) 5:e1068. doi: 10.1038/cddis.2014.44 24556683 PMC3944259

[32] Marani M Tenev T Hancock D Downward J Lemoine NR. Identification of Novel Isoforms of the BH3 Domain Protein Bim Which Directly Activate Bax To Trigger Apoptosis. Mol Cell Biol (2002) 22:3577–89. doi: 10.1128/mcb.22.11.3577-3589.2002 PMC133811 11997495

[33] Finetti F Patrussi L Masi G Onnis A Galgano D Lucherini OM. Specific Recycling Receptors are Targeted to the Immune Synapse by the Intraflagellar Transport System. J Cell Sci (2014) 127:1924–37. doi: 10.1242/jcs.139337 PMC4004972 24554435

[34] Patrussi L Capitani N Cattaneo F Manganaro N Gamberucci A Frezzato F. p66Shc Deficiency Enhances CXCR4 and CCR7 Recycling in CLL B Cells by Facilitating Their Dephosphorylation-Dependent Release From β-Arrestin at Early Endosomes. Oncogene (2018) 37:1534–50. doi: 10.1038/s41388-017-0066-2 29326436

[35] Liu Q Yin X Languino LR Altieri DC. Evaluation of Drug Combination Effect Using a Bliss Independence Dose–Response Surface Model. Stat Biopharm Res (2018) 10:112–22. doi: 10.1080/19466315.2018.1437071 PMC6415926 30881603

[36] Chou TC Talalay P. Quantitative Analysis of Dose-Effect Relationships: The Combined Effects of Multiple Drugs or Enzyme Inhibitors. Adv Enzyme Regul (1984) 22:27–55. doi: 10.1016/0065-2571(84)90007-4 6382953

[37] Xiao W Ando T Wang HY Kawakami Y Kawakami T. Lyn-And PLC-β3-Dependent Regulation of SHP-1 Phosphorylation Controls Stat5 Activity and Myelomonocytic Leukemia-Like Disease. Blood (2010) 116:6003–13. doi: 10.1182/blood-2010-05-283937 PMC3031387 20858858

[38] Bortner CD Cidlowski JA. Caspase Independent/Dependent Regulation of K+, Cell Shrinkage, and Mitochondrial Membrane Potential During Lymphocyte Apoptosis. J Biol Chem (1999) 274:21953–62. doi: 10.1074/jbc.274.31.21953 10419518

[39] Itchaki G Brown JR. The Potential of Venetoclax (ABT-199) in Chronic Lymphocytic Leukemia. Ther Adv Hematol (2016) 7:270–87. doi: 10.1177/2040620716655350 PMC5026291 27695617

[40] Rahmani M Nkwocha J Hawkins E Pei X Parker RE Kmieciak M. Cotargeting BCL-2 and PI3K Induces BAX-Dependent Mitochondrial Apoptosis in AML Cells. Cancer Res (2018) 78:3075–86. doi: 10.1158/0008-5472.CAN-17-3024 PMC5984704 29559471

[41] Ashkenazi A Salvesen G. Regulated Cell Death: Signaling and Mechanisms. Annu Rev Cell Dev Biol (2014) 30:337–56. doi: 10.1146/annurev-cellbio-100913-013226 25150011

[42] Carneiro BA El-Deiry WS. Targeting Apoptosis in Cancer Therapy. Nat Rev Clin Oncol (2020) 17:395–417. doi: 10.1038/s41571-020-0341-y 32203277 PMC8211386

[43] Cimmino A Calin GA Fabbri M Iorio MV Ferracin M Shimizu M. miR-15 and miR-16 Induce Apoptosis by Targeting BCL2. Proc Natl Acad Sci USA (2005) 102:13944–9. doi: 10.1073/pnas.0506654102 PMC1236577 16166262

[44] Fürstenau M Eichhorst B. Novel Agents in Chronic Lymphocytic Leukemia: New Combination Therapies and Strategies to Overcome Resistance. Cancers (Basel) (2021) 13:1–18. doi: 10.3390/cancers13061336 PMC8002361 33809580

[45] Stornaiuolo M La Regina G Passacantilli S Grassia G Coluccia A La Pietra V. Structure-Based Lead Optimization and Biological Evaluation of BAX Direct Activators as Novel Potential Anticancer Agents. J Med Chem (2015) 58:2135–48. doi: 10.1021/jm501123r 25668341

[46] Zhao G Zhu Y Eno CO Liu Y DeLeeuw L Burlison JA. Activation of the Proapoptotic Bcl-2 Protein Bax by a Small Molecule Induces Tumor Cell Apoptosis. Mol Cell Biol (2014) 34:1198–207. doi: 10.1128/mcb.00996-13 PMC3993561 24421393

[47] Xin M Li R Xie M Park D Owonikoko TK Sica GL. Small-Molecule Bax Agonists for Cancer Therapy. Nat Commun (2014) 5:4935. doi: 10.1038/ncomms5935 PMC4172359 25230299

[48] Liu G Yin T Kim H Ding C Yu Z Wang H. Structure-Activity Relationship Studies on Bax Activator SMBA1 for the Treatment of ER-Positive and Triple-Negative Breast Cancer. Eur J Med Chem (2019) 178:589–605. doi: 10.1016/j.ejmech.2019.06.004 31220676

[49] Vervloessem T Sasi BK Xerxa E Karamanou S Kale J La Rovere RM. BDA-366, a Putative Bcl-2 BH4 Domain Antagonist, Induces Apoptosis Independently of Bcl-2 in a Variety of Cancer Cell Models. Cell Death Dis (2020) 11:769. doi: 10.1038/s41419-020-02944-6 PMC7498462 32943617

[50] Zhang S Kipps TJ. The Pathogenesis of Chronic Lymphocytic Leukemia. Annu Rev Pathol (2014) 9:103–18. doi: 10.1146/annurev-pathol-020712-163955 PMC4144790 23987584

[51] Stevenson FK Forconi F Packham G. The Meaning and Relevance of B-Cell Receptor Structure and Function in Chronic Lymphocytic Leukemia. Semin Hematol (2014) 51:158–67. doi: 10.1053/j.seminhematol.2014.05.003 25048780

[52] Zou ZJ Fan L Wang L Xu J Zhang R Tian T. miR-26a and miR-214 Down-Regulate Expression of the PTEN Gene in Chronic Lymphocytic Leukemia, But Not PTEN Mutation or Promoter Methylation. Oncotarget (2015) 6:1276–85. doi: 10.18632/oncotarget.2626 PMC4359232 25361012

[53] Rizzo D Lotay A Gachard N Marfak I Faucher JL Trimoreau F. Very Low Levels of Surface CD45 Reflect CLL Cell Fragility, are Inversely Correlated With Trisomy 12 and Are Associated With Increased Treatment-Free Survival. Am J Hematol (2013) 88:747–53. doi: 10.1002/ajh.23494 23733486

[54] Motiwala T Majumder S Kutay H Smith DS Neuberg DS Lucas DM. Methylation and Silencing of Protein Tyrosine Phosphatase Receptor Type O in Chronic Lymphocytic Leukemia. Clin Cancer Res (2007) 13:3174–81. doi: 10.1158/1078-0432.CCR-06-1720 PMC3074612 17545520

[55] Suljagic M Longo PG Bennardo S Perlas E Leone G Laurenti L. The Syk Inhibitor Fostamatinib Disodium (R788) Inhibits Tumor Growth in the E - TCL1 Transgenic Mouse Model of CLL by Blocking Antigen-Dependent B-Cell Receptor Signaling. Blood (2010) 116:4894–905. doi: 10.1182/blood-2010-03-275180 20716772

[56] O’Hayre M Niederst M Fecteau JF Nguyen VM Kipps TJ Messmer D. Mechanisms and Consequences of the Loss of PHLPP1 Phosphatase in Chronic Lymphocytic Leukemia (CLL). Leukemia (2012) 26:1689–92. doi: 10.1038/leu.2012.6 PMC3395972 22237780

[57] Zonta F Pagano MA Trentin L Tibaldi E Frezzato F Trimarco V. Lyn Sustains Oncogenic Signaling in Chronic Lymphocytic Leukemia by Strengthening SET-Mediated Inhibition of PP2A. Blood (2015) 125:3747–55. doi: 10.1182/blood-2014-12-619155 25931585

[58] Cui B Chen L Zhang S Mraz M Fecteau JF Yu J. Micro RNA-155 Influences B-Cell Receptor Signaling and Associates With Aggressive Disease in Chronic Lymphocytic Leukemia. Blood (2014) 124:546–54. doi: 10.1182/blood-2014-03-559690 PMC4110661 24914134

[59] Negro R Gobessi S Longo PG He Y Zhang ZY Laurenti L. Overexpression of the Autoimmunity-Associated Phosphatase PTPN22 Promotes Survival of Antigen-Stimulated CLL Cells by Selectively Activating AKT. Blood (2012) 119:6278–87. doi: 10.1182/blood-2012-01-403162 PMC3383194 22569400

[60] Tsui FWL Martin A Wang J Tsui HW. Investigations Into the Regulation and Function of the SH2 Domain-Containing Protein-Tyrosine Phosphatase, SHP-1. Immunol Res (2006) 35:127–36. doi: 10.1385/IR:35:1:127 17003515

[61] Lemm EA Valle-Argos B Smith LD Richter J Gebreselassie Y Carter MJ. Preclinical Evaluation of a Novel SHIP1 Phosphatase Activator for Inhibition of PI3K Signaling in Malignant B Cells. Clin Cancer Res (2020) 26:1700–11. doi: 10.1158/1078-0432.CCR-19-2202 PMC7124891 31831562

[62] Dempke WCM Uciechowski P Fenchel K Chevassut T. Targeting SHP-1, 2 and SHIP Pathways: A Novel Strategy for Cancer Treatment? Oncol (2018) 95:257–69. doi: 10.1159/000490106 29925063

