# Supplementary material for: Glycerophosphoinositol Promotes Apoptosis of Chronic Lymphocytic Leukemia Cells by Enhancing Bax Expression and Activation
Source: Front Oncol. 2022 Mar 22;12:835290. doi: 10.3389/fonc.2022.835290 (PMC8980805; doi:10.3389/fonc.2022.835290)
Supplement: Supplementary file 1 [file DataSheet_1.pdf]

Supplementary Material

Supplementary Figures

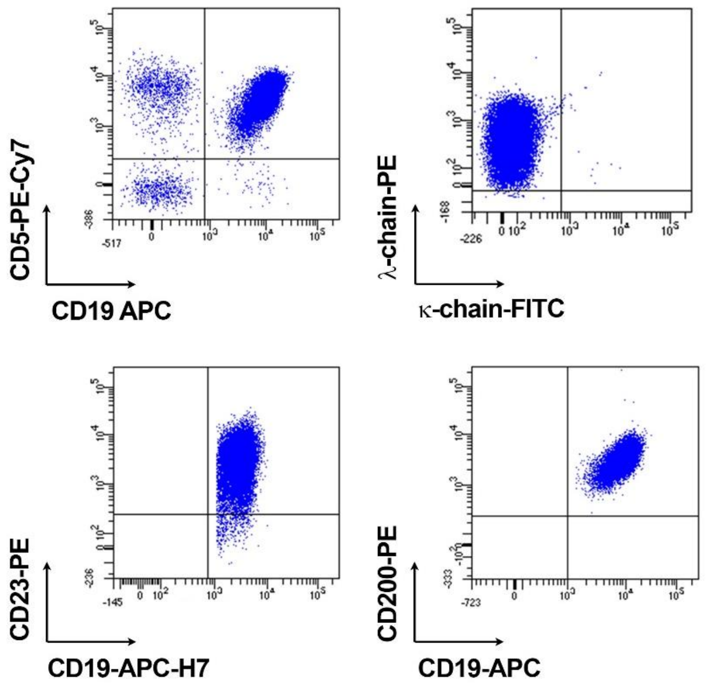

Supplementary Figure 1. Cytograms of a representative case of CLL. B lymphocytes analyzed ($CD19^+$) are positive to CD5, express one type of immunoglobulin light chain (λ), and are positive to CD23 and to CD200.

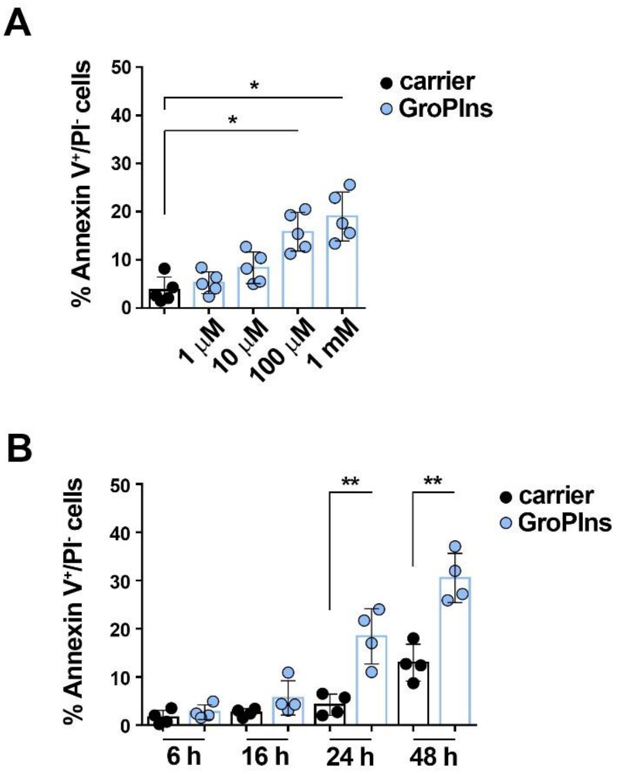

.

Supplementary Figure 2. Time course and dose-response treatments of CLL cells with GroPIns. Flow cytometric analysis of the percentages of Annexin V^+/PI^- cells in B lymphocytes purified from peripheral blood of CLL patients (CLL; **A**: n=5; **B**: n=4). Samples were treated with either carrier or GroPIns at the indicated concentrations at 37°C (**A**). The concentration of 100 μM was then used for time-course experiments (**B**). Mean±SD. Anova two-way test, Multiple Comparison. $p \leq 0.01$, **; $p \leq 0.05$, *.

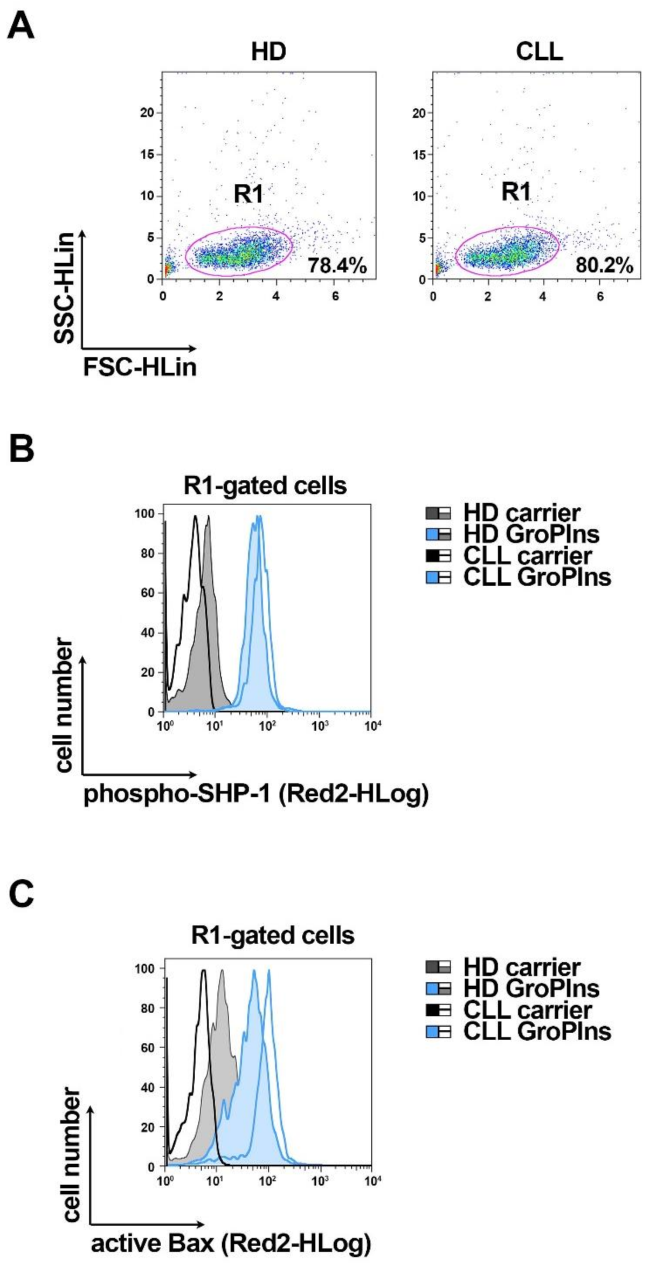

Supplementary Figure 3. Representative flow cytometric plots and histograms of phospho-SHP-1 and active Bax. Flow cytometric analysis of phospho-SHP-1 and active Bax in B cells purified from peripheral blood of healthy donors (HD; n=6) and CLL patients (CLL; n=6). Samples were treated with either carrier or 100 μM GroPIns at 37°C for either 30 min (phospho-SHP-1) or 20 min (active Bax). (**A**). Gating strategy. R1 represents live B lymphocytes. (**B, C**). Cells were stained with anti-phospho-SHP-1 (**B**) and active Bax (**C**) antibodies. Histograms show R1-gated cells from a representative HD and a representative CLL patient.

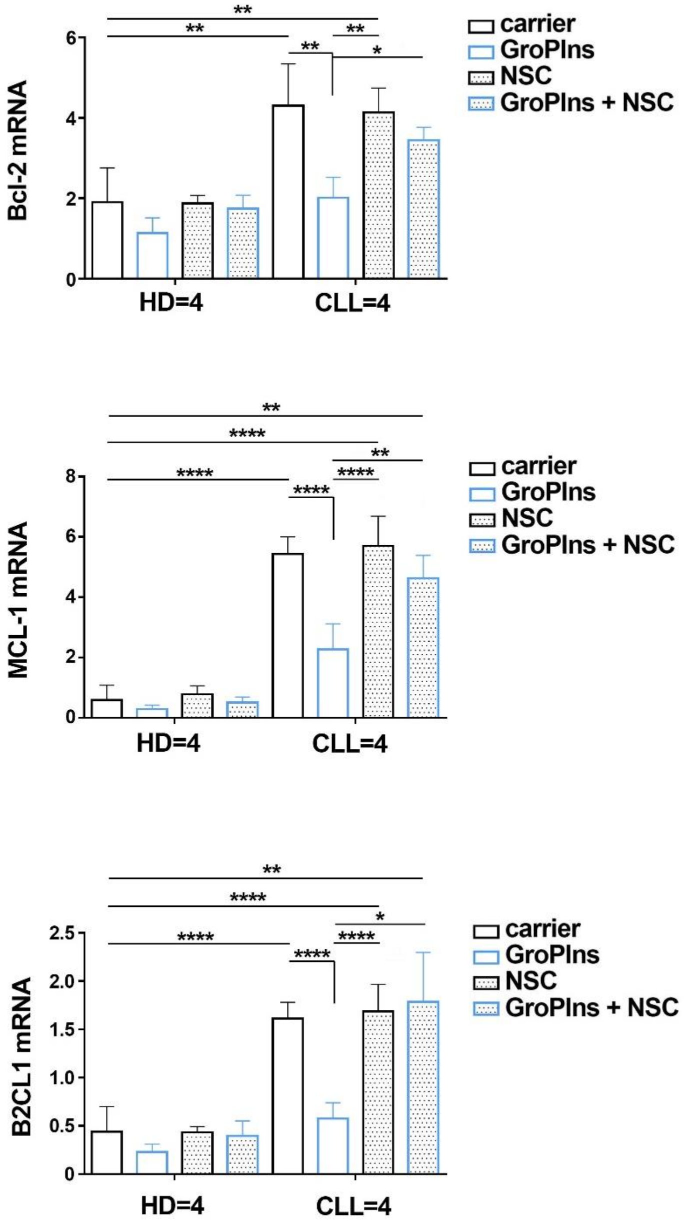

Supplementary Figure 4. GroPIns decreases the expression of Bcl-2, MCL-1 and B2CL2 in a SHP-1-dependent manner. Quantitative RT-PCR analysis of Bcl-2, MCL-1 and B2CL2 mRNA in B lymphocytes purified from peripheral blood of healthy donors (HD; n=4) and CLL patients (CLL; n=4) treated with either carrier or 100 μM GroPIns at 37°C for 24 h in the presence or absence of 50 μM NSC-87887 (NSC). The relative gene transcript abundance was determined on triplicate samples using the ddCt method and normalized to HPRT1. Mean±SD. Anova two-way test, Multiple Comparison. $p \leq 0.0001$, ****; $p \leq 0.01$, **; $p \leq 0.05$, *.

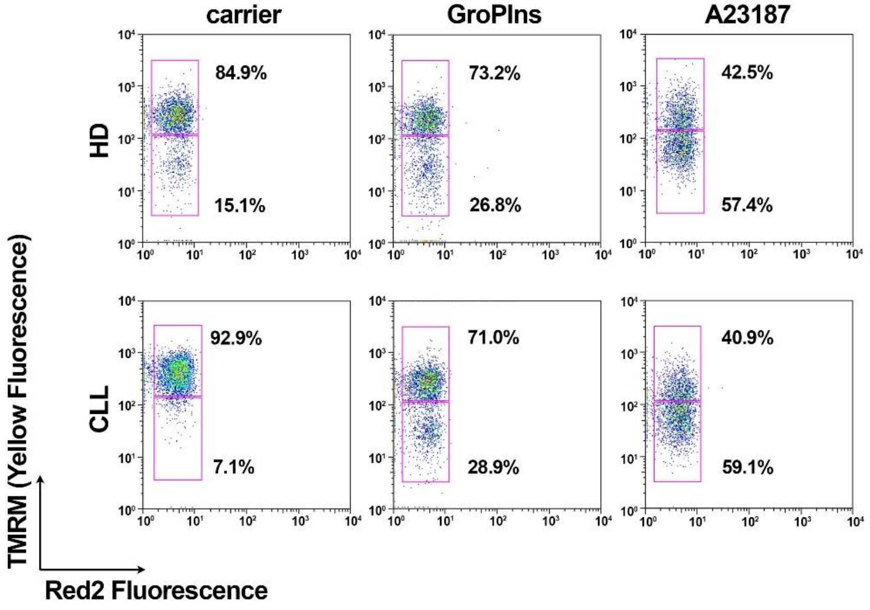

Supplementary Figure 5. Analysis of TMRM-loaded cells. Flow cytometric plots of B lymphocytes purified from peripheral blood of a representative healthy donor (HD) and a representative CLL patient (CLL) loaded with TMRM and then for 4 h at 37°C with either carrier or 100 μM GroPIns or 500 ng/ml A23187. Lower gates include $TMRM^{low}$ cells, upper gates include $TMRM^{high}$ cells.

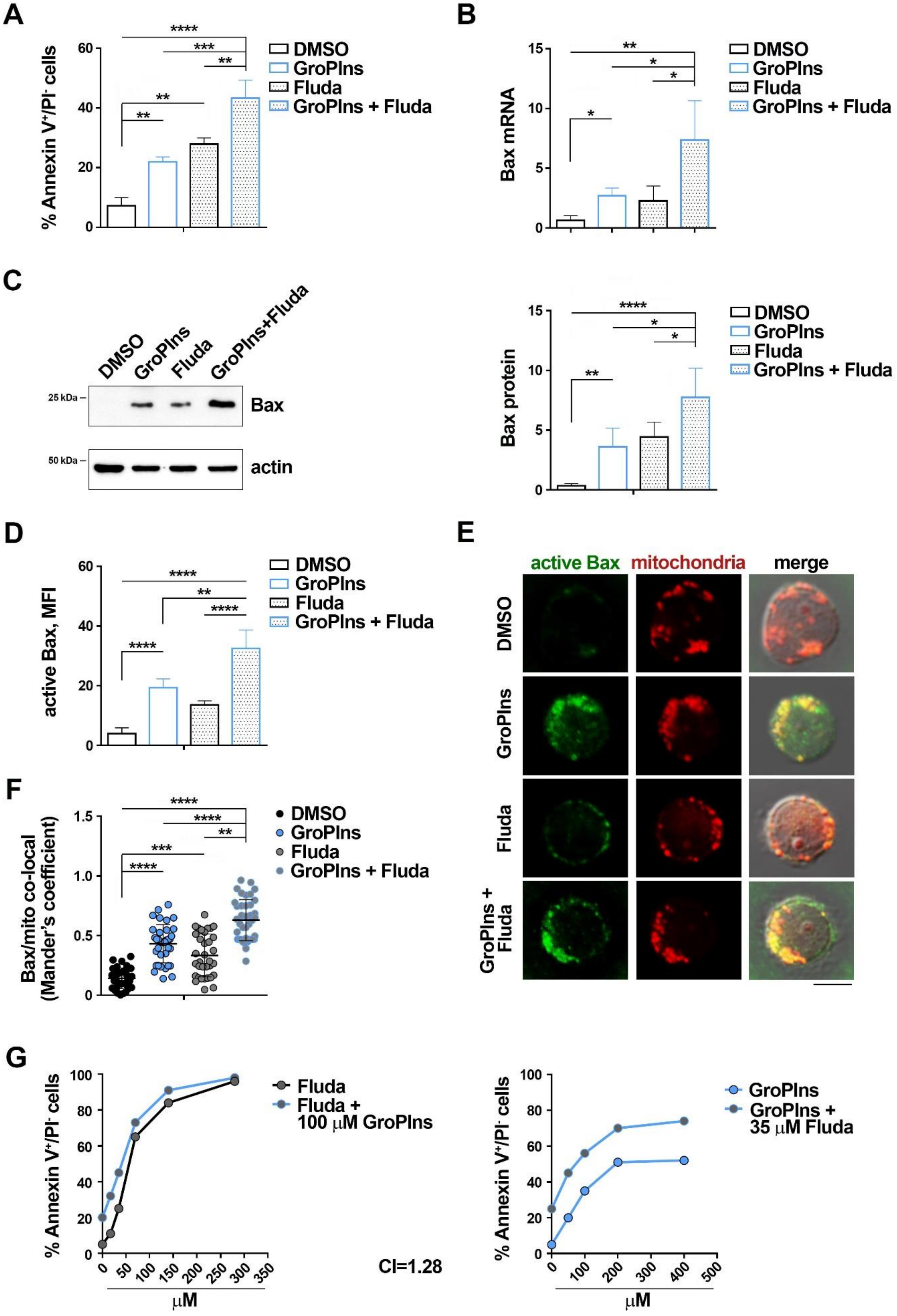
A
% Annexin V+/PI- cells
DMSO
GroPIns
Fluda
GroPIns + Fluda
B
Bax mRNA
C
DMSO
GroPIns
Fluda
GroPIns+Fluda
25 kDa
Bax
50 kDa
actin
Bax protein
D
active Bax, MFI
E
active Bax
mitochondria
merge
F
Bax/mito co-local
(Mander's coefficient)
G
Fluda
Fluda +
100 μM GroPIns
μM
CI=1.28
GroPIns
GroPIns +
35 μM Fluda

Supplementary Figure 6. GroPIns enhances the pro-apoptotic effect of Fludarabine in CLL cells. (**A**)**.** Flow cytometric analysis of the percentages of Annexin V^+/PI^- cells in B lymphocytes purified from peripheral blood of CLL patients (CLL; n=5) treated with either 100 μM GroPIns or 35 μM Fludarabine or the combination of both for 24 h at 37°C. (**B**). Quantitative RT-PCR analysis of Bax mRNA in B lymphocytes purified from peripheral blood of CLL patients (CLL; n=5) and treated as in (**A**). The relative gene transcript abundance was determined on triplicate samples using the ddCt method and normalized to HPRT1. (**C**). Immunoblot analysis with anti-Bax antibodies of postnuclear supernatants of B lymphocytes purified from peripheral blood of CLL patients (CLL; n=3) treated as in (**A**). The stripped filters were reprobed with anti-actin antibodies. Molecular weights (kDa) are indicated. The quantification of three independent experiments is shown on the right. (**D**)**.** Flow cytometric analysis of active Bax in B lymphocytes purified from peripheral blood of CLL patients (CLL; n=5) and treated for 20 min at 37°C with either 100 μM GroPIns or 35 μM Fludarabine or the combination of both. (**E**). Immunofluorescence analysis of active Bax (green) and mitochondria (Mitotracker) (red) in B lymphocytes purified from peripheral blood of CLL patients (CLL; n=3) treated as above. Immunofluorescence images were acquired on confocal microscope using 60 × objective. Representative immunofluorescence images are shown. Size bar, 5 μm. The quantification using Mander's coefficient of the weighted colocalization of active Bax with mitochondria in individual medial confocal sections is shown in (**F**). (**G**). Flow cytometric analysis of the percentages of Annexin V^+/PI^- cells in B lymphocytes purified from peripheral blood of a pool of 2 CLL patients treated with either GroPIns or Fludarabine or with the combination of both at the indicated concentrations for 24 h at 37°C. The calculated Cooperation Index (CI) is indicated. Mean±SD. Anova one-way test, Multiple Comparison. $p \leq 0.0001$, ****; $p \leq 0.001$, ***; $p \leq 0.01$, **; $p \leq 0.05$, *.

Supplementary Tables

Supplementary Table 1. List of primers used in this study.

	Primer Forward	Primer Reverse
**Bax**	GAGAGGTCTTTTTCCGACTGG	CCTTGAGCACCAGTTTGCTG
**Bcl-2**	GGAGGCTGGGATGCCTTT	CCAGATAGGCACCCAGGGT
**MCL-1**	GCTGGGAGTTGGTCGGGGA	TCGTAAGGTCTCCAGCGCCT
**B2CL1**	ATGAACTCTTCCGGGATGG	TGGATCCAAGGCTCTAGGTG
**HPRT1**	AGATGGTCAAGGTCGCAAG	GTATTCATTATAGTCAAGGGCATATC

Supplementary Table 2. Clinical parameters of CLL patients used in this study. IGHV: Immunoglobulin heavy variable chain; WBC: white blood cell count; Ly: lymphocytes.

CLL Patient	Mutational IGHV status	Karyotype	WBC (n/µl)	Ly % (n/µl)
**# 1**	mutated	wild-type	7.910	41.2
**# 2**	mutated	wild-type	17.590	65.2
**# 3**	mutated	13q	24.730	79.6
**# 4**	mutated	13q	12.900	66.2
**# 5**	mutated	13q	52.810	nd
**# 6**	mutated	wild-type	16.380	72.5
**# 7**	mutated	13q	10.770	67.8
**# 8**	mutated	11q	9.210	92.0
**# 9**	unmutated	13q	86.940	92.0
**# 10**	unmutated	13q	10.580	62.6
**# 11**	mutated	12+	83.750	69.2
**# 12**	unmutated	13q	47.830	97.0
**# 13**	mutated	11q	27.120	69.4
**# 14**	unmutated	17p	39.420	91.3
**# 15**	mutated	13q	32.960	93.0
**# 16**	unmutated	11q	69.100	81.6
**# 17**	unmutated	17p	19.900	70.3
**# 18**	unmutated	wild-type	113.700	96.6
**# 19**	unmutated	12+	35.190	73.6
**# 20**	unmutated	13q	58.120	88.2
**# 21**	unmutated	17p	74.660	90.6
**# 22**	unmutated	13q	79.450	95.6
**# 23**	unmutated	13q	39.070	95.5
**# 24**	mutated	13q	36.850	91.7
**# 25**	mutated	13q	22.450	77.7
**# 26**	unmutated	13q	206.800	98.5
**# 27**	unmutated	12+	65.700	86.2
**# 28**	mutated	13q	30.000	83.7
**# 29**	mutated	13q	66.400	88.7
**# 30**	unmutated	11q	121.900	96.0
**# 31**	mutated	13q	198.000	97.0
**# 32**	unmutated	11q	106.100	94.5
**# 33**	unmutated	12+	66.900	93.3
**# 34**	mutated	13q	88.230	90.9
**# 35**	unmutated	11q	124.900	95.9
**# 36**	mutated	wild-type	26.680	83.9
**# 37**	mutated	13q	15430	81.4
**# 38**	mutated	wild-type	43600	82.6
**# 39**	unmutated	17p	23600	67.8
**# 40**	mutated	wild-type	69740	94.0
